# Hybrid plasmonic valley-Hall topological insulators

**DOI:** 10.1515/nanoph-2023-0902

**Published:** 2024-04-15

**Authors:** Sam Lin, Zi Jing Wong

**Affiliations:** Department of Materials Science and Engineering 14736 , Texas A&M University, College Station, TX 77843, USA; Department of Aerospace Engineering, Texas A&M University, College Station, TX 77843, USA; School of Electronic Science and Technology, Eastern Institute of Technology, Ningbo, Zhejiang 315200, China

**Keywords:** photonic topological insulators, light–matter interaction, plasmonics, integrated photonics

## Abstract

The emerging field of photonic topological insulators offers promising platforms for high-performance optical communication, computing, and sensing. However, conventional photonic topological insulator designs typically operate within the diffraction limit due to their dielectric nature. This limitation imposes constraints on device miniaturization, reduces light–matter interaction, and decreases overall device sensitivity. Introducing a new valley-Hall hybrid plasmonic topological insulator, we overcome this limitation by exploiting the coupling of surface plasmon oscillations with the optical modes of a dielectric photonic crystal, allowing for sub-diffraction vertical confinement of light. Deep-subwavelength chiral edge states can, therefore, be generated and robustly guided along disordered Z-shaped topological boundaries with much lower propagation loss compared to purely plasmonic platforms. Such extreme manipulation of light on an integrated chip platform maximizes light–matter interaction and opens the door for truly compact and efficient optical modulators, molecular sensors, and next-generation nanophotonic and quantum devices.

## 1 Introduction

The study of photonic topological insulators is a quickly growing field in photonics and optics, sparked by the translation of mathematical concepts from the world of electronic topological insulators. This revolution in photonics is fueled by the discovery of new principles for manipulating light based on band topology, such as topological band gaps, robust topological waveguiding, and localization [1], [2], [3], [4], [5], [6], [7], [8]. Photonic topological phenomena have been realized both theoretically and experimentally in periodic arrays of optical ring resonators [9], waveguide arrays [5], bianisotropic metamaterials [6], and photonic crystals [4], [7], [8]. Among these platforms, photonic crystals have emerged as candidates for realizing easily fabricable, compact, and fully integrated flat-optical devices. Importantly, the study of valley-Hall photonic crystals, which are compact and avoid the use of magnetic materials [10], have uncovered optical structures that realize robust delay lines [8], routing of valley-chirality-locked edge states [11], [12], optical switching [13], and lasing [14]. However, the dielectric composition of such topological structures constrains them to operate within the diffraction limit, which prevents device miniaturization and leads to low energy density and weak light–matter interaction.

To address this challenge, several works have proposed the design of topological plasmonic devices for deeply subwavelength electric field confinement and nanoscale light matter interaction [15], [16], [17]. A planar array of silver nanoparticles has been shown in simulation to host the quantum spin-Hall effect, supporting chiral edge states that propagate between topologically distinct domains [18]. Despite possessing tight electromagnetic confinement that beats the diffraction limit, the quantum spin-Hall mechanism requires an expanded unit cell, doubling device footprint, and is also based on a hard to fabricate nanosphere array, posing a barrier to chip-scale integration. Recently, a valley-Hall device based on metallic disks has been demonstrated [19], confining light to within a 30 nm thickness. Nevertheless, purely plasmonic topological devices in general inevitably exhibit severe plasmon damping that reduces photon lifetime within the desired light–matter interaction volume. As a result, additional sensitivity afforded by increased confinement is offset by electric field attenuation.

Here, we overcome the limitations of plasmonic topological insulators and propose a new valley-Hall hybrid plasmonic topological insulator. We achieve vertical confinement of electromagnetic energy beyond the diffraction limit by coupling surface plasmon oscillations with the optical modes of a dielectric photonic crystal slab. We then incorporate valley-Hall topology and design topological domains of such a hybrid plasmonic structure to generate and guide topologically protected edge states that demonstrate chirality and reduced ohmic losses compared to purely plasmonic waveguides. These characteristics allow us to achieve stronger light–matter interaction than conventional dielectric platforms, while preserving optical functionality useful for applications such as on-chip topological lasing and topological-waveguide based modulation and sensing. Finally, we validate topological robustness with 3D simulations and quantify propagation characteristics and robustness. Our findings lay out a path toward compact, low-loss topological plasmonic devices for next-generation integrated photonics.

## 2 Results and discussion

**Figure 1:**
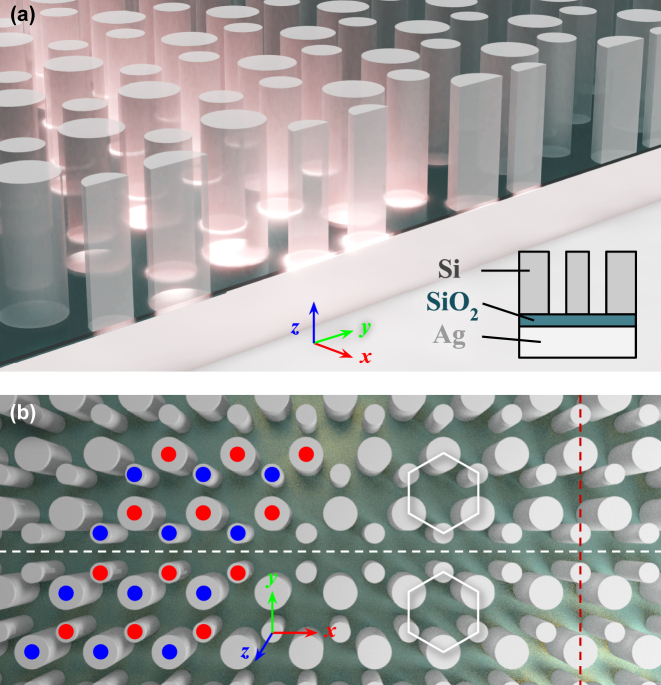
Structure of a valley-Hall hybrid plasmonic topological insulator (HPTI). (a) The HPTI is composed of a thin low-index SiO_2_ sandwiched between a Si nanopillar lattice and an optically thick silver metal film. Light propagates along topological boundaries as illustrated by a pink glow, and inset is a diagram of the nanostructure cross section, not to scale. (b) Compared to a structure with uniform rod diameters, breaking the inversion symmetry of each unit cell in reversed directions across the white dashed line creates two inequivalent topological domains. In the top domain, the rods of one triangular sublattice (blue) have reduced radii while the rods of the other (red) have increased radii, and the opposite is true for the bottom domain. The lattice constant is *a*_0_ = 480 nm. The radii of narrower and wider silicon rods are 0.165*a*_0_ and 0.235*a*_0_, respectively, and their heights are *a*_0_. The cross section taken in (a) is marked by red vertical dashes.

We begin the design of our hybrid plasmonic topological insulator, shown in Figure 1(a), by constructing a hexagonal lattice of dielectric nanopillars surrounded by air, producing the *C*_6*v*_ symmetric photonic analog of graphene. This structure can be described as two superimposed triangular sublattices, which we mark by red and blue dots in Figure 1(b). Conventional photonic topological insulators feature electric fields confined in high refractive index dielectrics, which hampers desired light matter interactions in surrounding low-refractive index materials. To address this, a metal substrate is introduced to our nanopillar photonic graphene, separated from nanopillars by a nanoscale gap layer of low-index dielectric. A large increase in electric field strength is thus observed for certain modes in the gap layer, which we attribute to two reinforcing effects. First, due to the presence of the substrate, the transverse magnetic (TM) modes of the nanopillar photonic graphene now couple to plasma oscillations within the metallic layer, which are highly confined surface plasmon polaritons. The resulting hybridized eigenmodes are spatially localized toward the metallic–dielectric interface, which drives electric fields into the gap layer. Second, the discontinuous step at the interface of the high- and low-index dielectrics induces an enhanced normal electric field in the nanoscale dielectric gap, enhancing coupling to TM surface plasmon oscillations. Thus, field enhancement can be improved by the choice of a high refractive index contrast material system, and we choose the high refractive index silicon (Si) and the low refractive index silica (SiO_2_) as our nanopillar and dielectric gap material, respectively. The combined interaction of these plasmonic and dielectric effects can be interpreted as the capacitor-like storage of electromagnetic energy between the polarization charge created at the dielectric interface and the plasma oscillations [20]. We note that the low index material can be partially replaced with materials with even lower refractive index materials such as liquid solutions for enhanced sensing of solute species (part 4, Supplementary). Finally, to enhance the lifetime and quality factor of hybrid plasmonic oscillations, we choose silver for our metallic substrate.

**Figure 2:**
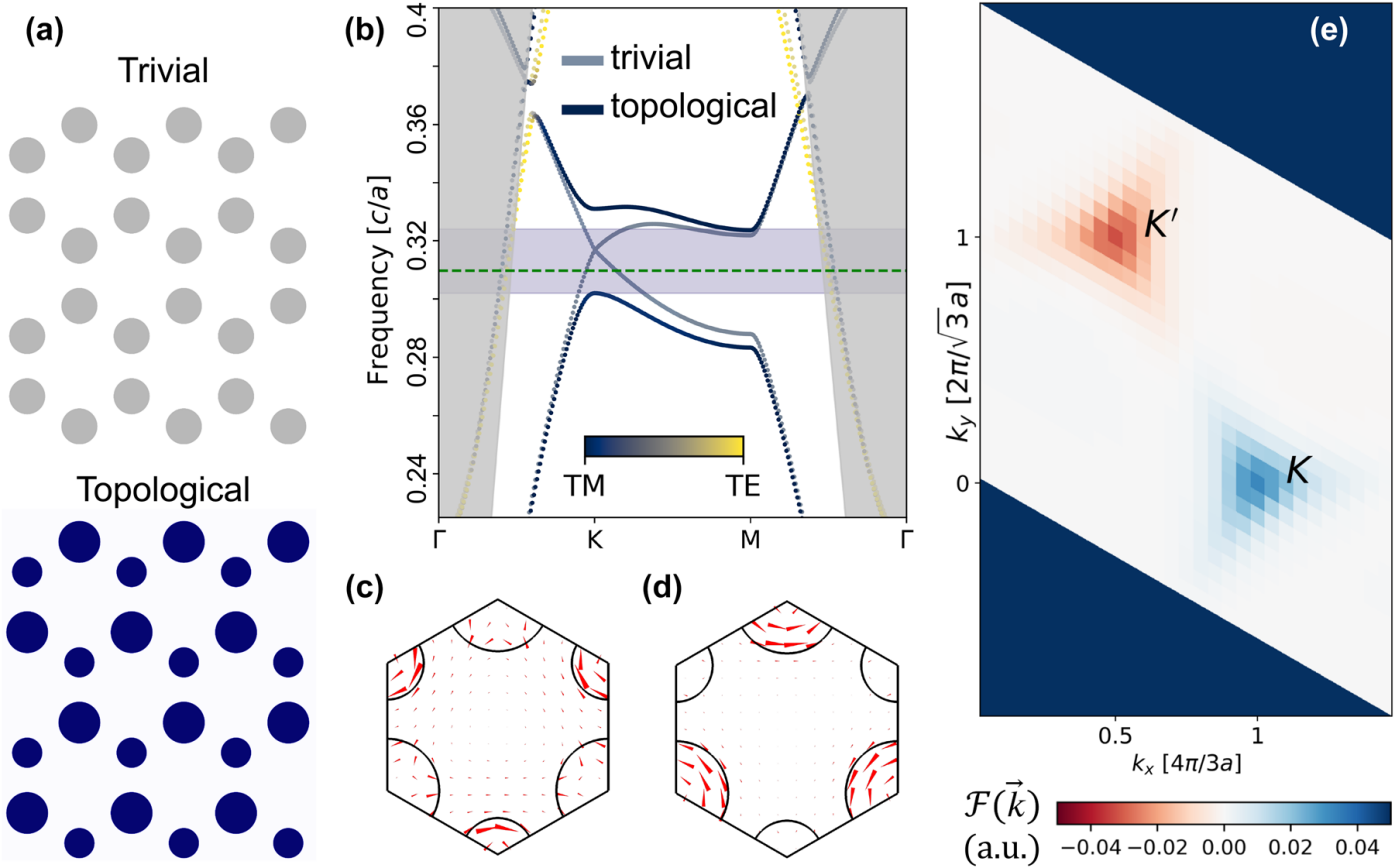
Band topology of a HPTI. (a) A top view illustration shows the effect of a *C*_3*v*_ symmetric perturbation applied to high refractive index cylinders. (b) Starting with the band structure of the topologically trivial photonic crystal (bands with muted colors), the structural perturbation lifts the degeneracy Dirac cone degeneracy at the K valleys, and the corresponding valley-Hall (blue and yellow bands) band structure features a topological band gap (purple). The color scale denotes the polarization character of eigenstates, the gray region denotes the light cone, and the dotted green line marks 1550 nm. (c, d) *C*_3*v*_ symmetric unit cells support (c) left- and (d) right-circularly polarized states at the K valleys, which contain counter-circulating Poynting vector distributions (red arrows). (e) The Berry curvature is computed on a discretized rhombic *k*-space unit cell, where opposite signs of the Berry curvature are seen at K and K′.

Next, we explore the design of a hybrid plasmonic topological band gap through modifying the symmetries of the lattice. The *C*_6*v*_ symmetry of photonic graphene creates two-fold degeneracies at Brillouin zone corners K and K′ with corresponding Dirac cone dispersion in the band structure (Figure 2(a)). Due to the underlying *C*_3*v*_ symmetry of the unit cell, doubly degenerate eigenstates at K and K′ can be chosen such that rotation by *R*_3_ has the effect of multiplying by a phase factor e^±2π*i*/3^, allowing the labeling of such eigenstates as right-circularly polarized (RCP) and left-circularly polarized (LCP). To realize a valley-Hall topological insulator, we break the inversion symmetry of a *C*_6*v*_ symmetric crystal with a *C*_3*v*_ symmetric perturbation and lift the degeneracy at K and K′. Here, such a structural perturbation is produced by increasing nanopillar diameters of one sublattice and decreasing those of the other sublattice. The resulting “valleys” formed by the lifted Dirac degeneracies contain RCP and LCP states localized within one set of sublattices (Figure 2(b) and (c), respectively).

Guided by our material choice, we designed the geometric parameters targeting a fabricable model photonic structure that attains a clear topological band gap around 1550 nm (part 1, Supplementary). While the starting pillar radius *r*_0_ = 0.020 ⋅ *a*_0_ was selected to minimize nanostructure aspect ratio, the pillar height of *h* = *a*_0_ was chosen as the minimum value where a large band gap emerges for the perturbed structure. Finally, the period of the nanopillar lattice was selected to be *a*_0_ = 480 nm so that the appropriate frequency is scaled to the position of the topological band gap.

To uncover the valley-Hall topology of our hybrid plasmonic nanopillar lattice, we evaluate its topological invariants through finite element calculations. Eigenmode field profiles are used to calculate and plot the Berry curvature $F\left(\vec{k}\right)$ over the first Brillouin zone. As shown in Figure 2(d), the Berry curvature is antisymmetric with respect to inversion of reciprocal space, and the integral of the Berry curvature over the entire Brillouin zone vanishes, in line with time reversal symmetry of the unit cell structure. Defining the valley Chern number as the integral of the Berry curvature over the triangular half Brillouin zone (HBZ) around K (K′), $C_{V}^{K\left({\text{K}}^{\prime }\right)}=\frac{1}{2\pi }\int_{\text{HB}{\text{Z}}_{\text{K}\left({\text{K}}^{\prime }\right)}}F\left(\vec{k}\right)d^{2}\vec{k}$ , one obtains $C_{V}^{\text{K}\left({\text{K}}^{\prime }\right)}\to \pm \frac{1}{2}$ for an infinitesimal *C*_3*v*_ perturbation. In such cases, Berry curvature distribution is strongly localized at K and K′. However, for finite perturbation, as is required to create a sufficiently large band gap, the Berry curvature distribution broadens, and Berry curvature from K′ and K leaks into the opposite half Brillouin zone. The result is that $C_{V}^{\text{K}\left({\text{K}}^{\prime }\right)}$ decreases in magnitude from its small-perturbation limit [21], [22], [23]. Nevertheless, topologically protected edge states exist due to the sign change of the valley-Chern number. To exploit the topological gap for near-infrared operation, we tune the physical parameters of the hybrid plasmonic topological insulator to shift the degenerate Dirac point wavelength to near 1550 nm (Figure 2(a)). The lattice parameter is chosen to be *a*_0_ = 480 nm, and silicon rods are assigned radius *r* = 0.2 ⋅ *a*_0_ and height *h*_Si_ = *a*_0_.

**Figure 3:**
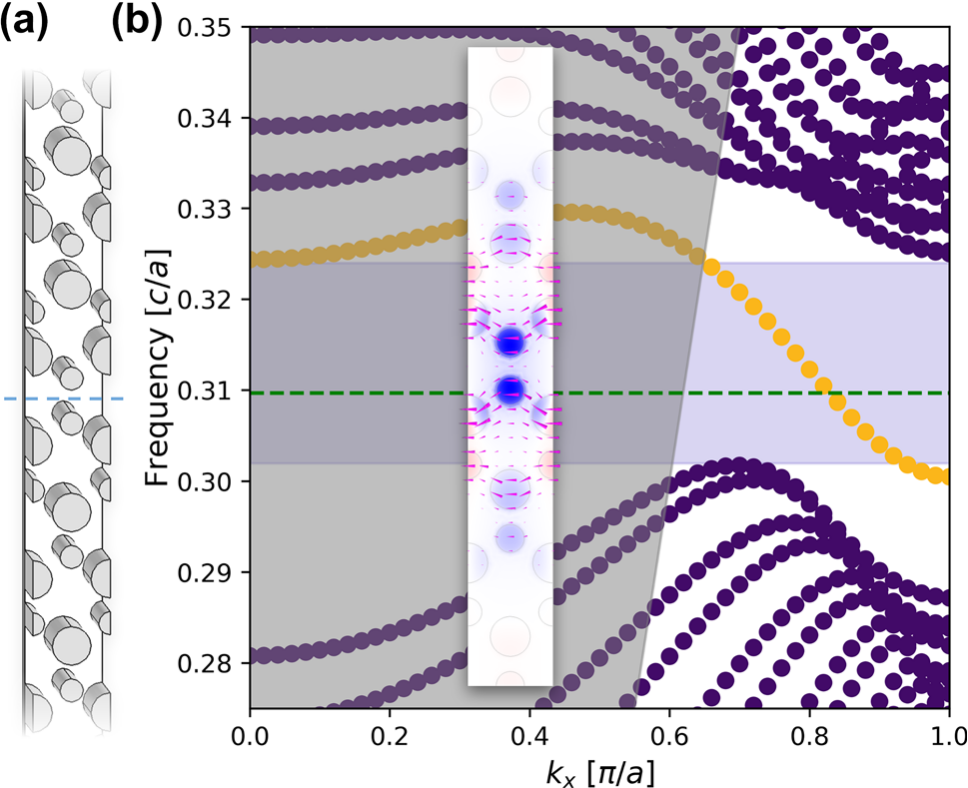
Dispersion of an edge state generated on the HPTI domain boundary. (a) A unit-cell-wide vertical slice of the photonic crystal containing a topological domain boundary (blue dashes) is depicted and constructed as a simulation domain. (b) The topological edge state (yellow) lies between transverse magnetic bulk bands (purple) separated by a band gap (shaded purple) from 0.302 to 0.324 in units of normalized frequency. Within the band gap, the edge state dispersion avoids the light cone (shaded gray), where propagating modes are lossy. Considering 1550 nm (green dashes), the inset shows *E*_*z*_ (color plot) and time averaged Poynting vectors (magenta) of the topological edge state within the low index dielectric layer.

To realize valley-Hall edge states, the symmetry lowering perturbation explained in the previous section is introduced differently to two regions of the hybrid plasmonic crystal (Figure 1(b)). Specifically, the sign of the dielectric perturbation is reversed across topological domains. In one domain, we enlarge the silicon rod diameters of one sublattice (blue/red in Figure 1(b)) and shrink those of the other sublattice (red/blue in Figure 1(b)), while the opposite is done within the other domain. The result is two distinct topological domains separated by a boundary on which the bulk-boundary correspondence dictates the existence of a pair of counter-propagating boundary states. Here, a zigzag-type topological domain boundary is designed such that the nanopillars nearest to the boundary are smaller in diameter to further reduce the volume of light–matter interaction. To numerically demonstrate the existence of these states, we calculate the projected band structure of the two-domain hybrid plasmonic topological insulator. Figure 3(a) shows a diagram of the considered structure, which is a lattice-constant-wide supercell straddling the topological boundary. The eigenstates of this hybrid plasmonic structure were then computed with the finite element method (FEM) in COMSOL Multiphysics and plotted Figure 3(b), omitting the mirror image negative-*k*_*x*_ Brillouin zone region. Between the continua of states propagating within the crystal bulk, there exists an isolated band with its useful frequency dispersion lying under the light cone. Upon inspection, the field distributions of this set of modes are confined and localized near the innermost cylinders, which confirms their topological nature.

**Figure 4:**
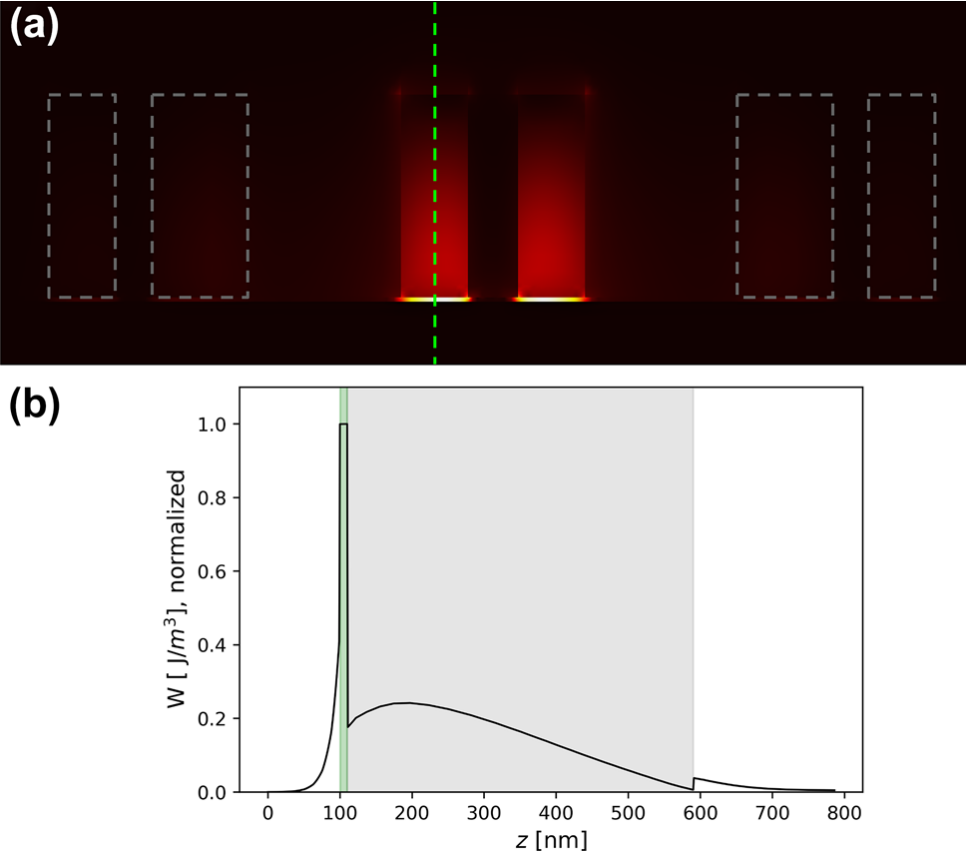
Extreme confinement of HPTI edge states. (a) Energy density of the topological edge state at 1550 nm sampled on the same cross section as in Figure 1, simultaneously exhibiting topological confinement to the topological domain boundary and plasmonic confinement to the 10 nm low-index SiO_2_ film. Si nanopillars located farther into the bulk are outlined in gray dashed lines for visibility. (b) Energy density trace taken along the axis (green dashes) in (a) of a central silicon nanopillar, showing enhanced electric field strength within the 10 nm SiO_2_ region (shaded green). This effect arises from the coupling of Si (shaded gray) dielectric modes with plasma oscillations within plasmonic silver (*z* < 100 nm).

Here, we examine the field confinement characteristics of the topological boundary waveguide in more detail. A cross-sectional view is made of the domain boundary along the same plane as in Figure 1, and we inspect computed electric fields within the supercell structure. Figure 4(a) shows a heat map of the electromagnetic energy density. A hybrid plasmonic topological waveguide confines light both laterally, in the form of topological localization, and vertically, by coupling dielectric resonances into charge density oscillations within the metallic substrate. To quantify the extent of the latter contribution, Figure 4(b) samples the energy density along the axis of the innermost pair of Si nanopillars and shows that the peak energy density occurs within the low-refractive index dielectric layer. Hence, compared to surrounding nanopillar and metal structures, hybridization with plasmonic surface modes creates energy density within the 10 nm gap far exceeding that of any other region. Such a degree of optical confinement within a thickness of only 10 nm overcomes the diffraction limit barrier. Not only will molecular-scale interaction with light lead to high performance chip-based sensors but the concept will also enhance the capabilities of thin optoelectronic materials such as carrier modulated conducting oxides, quantum wells, and novel 2D material systems. From numerical results, 7.3 % of the propagating wave’s electromagnetic energy is concentrated inside the low index dielectric gap. More detailed analysis on the subwavelength energy confinement and enhanced sensing capability can be found in the Supplementary Materials (part 4).

**Figure 5:**
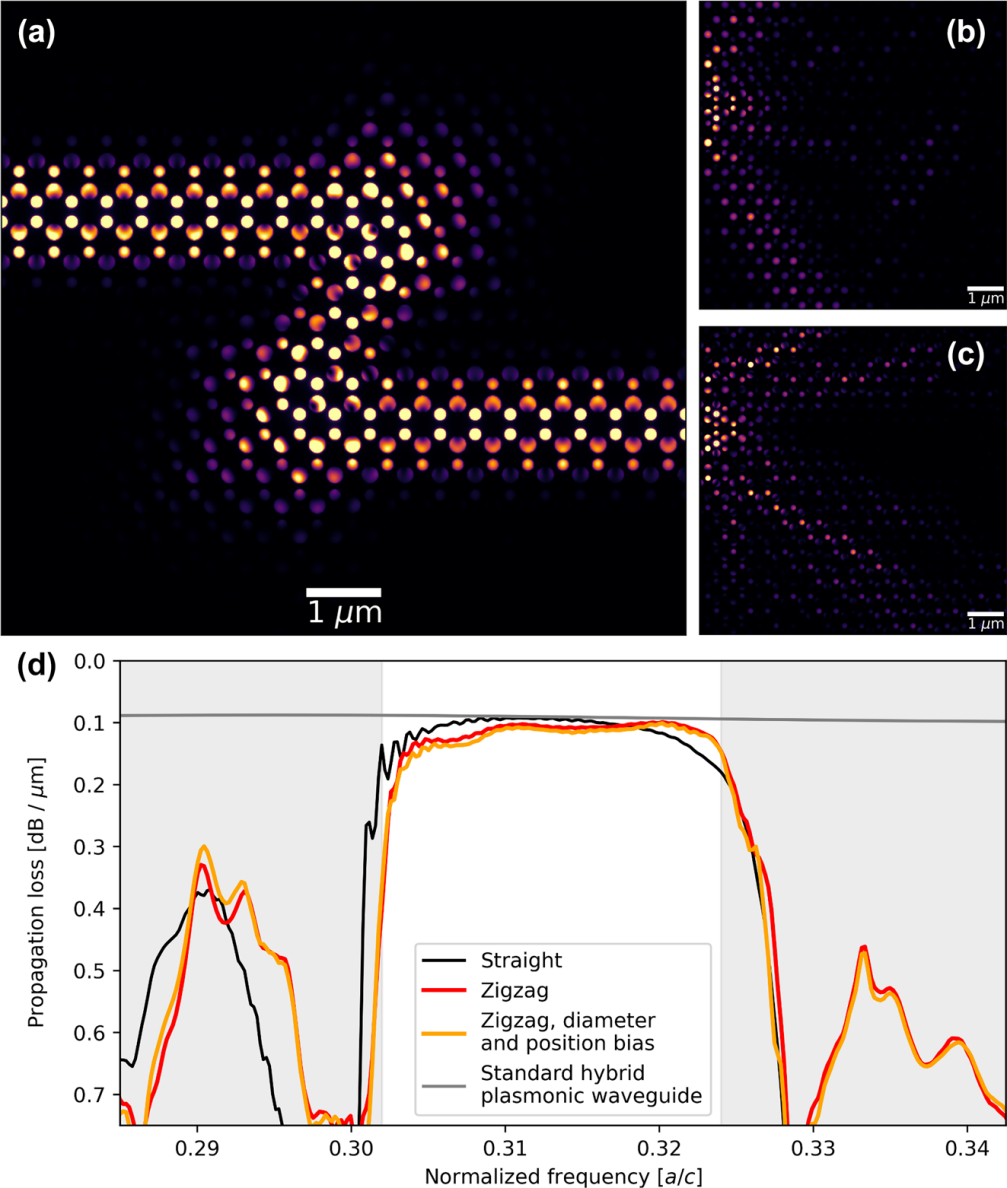
Robust propagation of HPTI edge states. (a–c) Energy densities within the low refractive index layer of a Z-shaped topological waveguide under continuous wave optical excitation. (a) Within the band gap, light is guided along a sharp topological domain boundary with two 120° bends, while (b, c) at other frequencies, light predominantly couples into bulk, unguided modes. Excitation wavelengths were (a) 1550 nm, (b) 1640 nm, and (c) 1440 nm, respectively. (d) Straight, Z-shaped, and disordered Z-shaped domain boundaries all exhibit propagation loss levels of approximately 0.1 dB μm^−1^ over a broad frequency range. Disorder was introduced by slightly perturbing the position and diameters of the silicon rods. Gray regions indicate frequency range outside the topological gap, in which propagation loss suffers due to the coupling of energy into bulk states. The gray curve indicates the transmittance of a straight standard hybrid plasmonic waveguide, which exhibits similar propagation loss compared to the topological transmission window.

Valley-Hall systems endow boundary states with topological protection against certain classes of perturbations. An important result is that boundary states within the band gap may propagate through sharp bends in domain boundary [10], [11], [12], [14], [21], [24], and we show here that this robustness is preserved for a valley-Hall hybrid plasmonic crystal, even in the presence of plasmonic losses. To do so, the dielectric profile of the three-dimensional structure was built and simulated in Lumerical FDTD. The topological edge state was excited using rotating dipoles within the low index dielectric layer on the left boundary of Figure 5(a)–(c). Transmitted power was measured after a waveguide length of 30*a*_0_. We account for imperfect absorbing simulation boundaries by introducing material absorption in the outermost lattice structure to eliminate back-reflection. Figure 5(a) shows the electric field distribution within the 10 nm layer of SiO_2_, illustrating the robust propagation of light through two sharp waveguide bends. On the other hand, optical excitations at wavelengths away from the topological gap are coupled to delocalized, unguided bulk modes. Figure 5(b) and (c) demonstrate this behavior at 1640 nm and 1440 nm, respectively. To quantify these results, we examine the transmittance spectrum of a straight topological waveguide and that of one with two sharp bends. To conserve the zigzag-type domain boundary, waveguide bends are made to be 120°. As shown in Figure 5(d), propagation loss reaches a minimum of 0.09 dB μm^−1^ for both boundary shapes and is better than 0.15 dB μm^−1^ over an 88 nm range covering the C band. Additionally, we show that such a waveguide is robust to structural disorders by introducing ±10 % perturbations in nanopillar diameter and up to 20 nm deviations in nanopillar position within the diagonal-running length of the Z-shaped topological waveguide. Introducing such deviations from ideal structure induces an additional propagation loss of merely ∼0.01 dB μm^−1^. Finally, we compare the propagation loss of this topological waveguide with that of a rectangular ridge-type plasmonic waveguide. Within the topological band gap, our topological waveguide geometries achieve a similar propagation distance as a rectangular hybrid plasmonic waveguide with a 10 nm silica gap, a width of 160 nm, and a height of 320 nm that maintains a propagation loss level of 0.09 dB μm^−1^. Thus, HPTI waveguiding allows for lower-loss robust chip-scale signal transport compared to conventional plasmonic platforms.

## 3 Conclusions

Although photonic topological insulators have been studied extensively in the past, silicon-on-insulator (SOI) and other dielectric platforms have become the default basis of fabrication for systems operating in the near-infrared. Such platforms lack the capability for deep-subwavelength optical confinement, which enlarges the footprints of integrated optical devices and reduces the scope of applications. In this letter, we have proposed valley-Hall hybrid-plasmonic topological insulators for sub-diffraction vertical confinement of light by coupling dielectric topological edge states to the highly localized fields of surface plasmon oscillations. Such confinement allows for enhanced light–matter interaction compared to conventional SOI platforms, while still being structurally simple, CMOS-compatible, and thus easily fabricable on-chip. Moreover, by exploiting the coupling between lossless dielectric resonances, the propagation length of topological hybrid plasmonic waveguides has been shown to be much lower than previous topological plasmonic systems. Our proposed design was realized by biasing the diameters of a silicon nanopillar photonic crystal suspended above a silver film by a thin layer of silica, but this baseline design can easily be generalized. Replacing the nanoscale dielectric layer, tunable materials such as transparent conducting oxides can be exploited to realize optical modulators, and sensors can be constructed by incorporating chemically sensitive polymeric materials for highly localized nanoscale detection. Even stronger optical confinement can be achieved with high refractive index materials such as germanium and gallium arsenide, opening the door toward the fabrication of ultralow threshold topological lasers.

## Supplementary Material

Supplementary Material Details
