# Supplementary material for: Hybrid plasmonic valley-Hall topological insulators
Source: Nanophotonics. 2024 Apr 15;13(15):2811–7. doi: 10.1515/nanoph-2023-0902 (PMC11501407; doi:10.1515/nanoph-2023-0902)
Supplement: Supplementary file 1 — Supplementary Material Details [file j_nanoph-2023-0902_suppl_001.pdf]

Supplementary Materials for:

Hybrid Plasmonic Valley-Hall Topological Insulators

Sam Lin[1] *and Zi Jing Wong*[1,2,3]*

[1] Department of Materials Science and Engineering, Texas A&M University, College Station, Texas 77843, USA

[2] Department of Aerospace Engineering, Texas A&M University, College Station, Texas 77843, USA

[3] School of Electronic Science and Technology, Eastern Institute of Technology, Ningbo, Zhejiang 315200, China

*Corresponding author. E-mail: zijing@tamu.edu

Contents

1. **Design of Structural Parameters for Hybrid Plasmonic Topological Insulators**
2. **Numerical Demonstration of the Transition Between Dielectric TM Modes and Hybrid Plasmonic Modes**
3. **Comparison with All-Dielectric and Pure-Plasmonic Topological Insulators**
4. **Analysis of Energy Confinement in Low Refractive Index Spacer**
5. **Numerical Demonstration of Chemical Sensing in Nanoscale Hybrid Plasmonic Gap Layer**
6. **Discussion of real-world fabrication and characterization studies**

1. Design of Structural Parameters for Hybrid Plasmonic Topological Insulators

Here we show the results of sensitivity analysis of the topological band gap with respect to the structural parameters a_0, h, and $r_{1,2}$. Starting with the model structure in the main text as a baseline, these parameters were modulated to show their influence on the topological band gap position. Due to the finite element eigenfrequency calculations returning spurious eigenmodes that decay quickly into the scattering boundary, we discriminate these modes from localized eigenmodes by calculating their Q factor. Lower Q factor modes are lighter shades of gray.

Fig. S1 shows the modulation of the scale factor a', which proportionally scales lattice constant, pillar height, and radius as $a_0 = a' \cdot 480$ nm, $h = 1.0 \cdot a_0$, and $r_{1,2} = (0.2 \pm 0.035) \cdot a_0$. The gap size of 10 nm was held constant, and a' was modulated from 0.4 to 1.6. The band structure remains qualitatively identical up to a frequency shift according to $f \propto 1/a'$.

Fig. S2 shows unit cell band structures for pillar heights $h = h' \cdot a_0$, where the scale factor h' was swept over values $h' = \{0.6, 0.8, 0.9, 1.1, 1.2, 1.4\}$. Higher aspect ratio nanopillars exhibit lower band gap frequencies and larger band gap openings.

Fig. S3 shows the effect of modulating pillar radius as $r_{1,2} = r' \cdot (0.2 \pm 0.035) \cdot a_0$, with scale factor r' swept from 0.7 to 1.3. A frequency shift is observed due to the change in the average refractive index within the patterned layer. On the other hand, a closing of the topological gap is observed at large nanopillar radii due to proximity between shrunken and expanded nanopillars. Circularly polarized eigenstates localized in one set of nanopillars are perturbed by the opposite set of nanopillars, reducing the contrast in effective mode index.

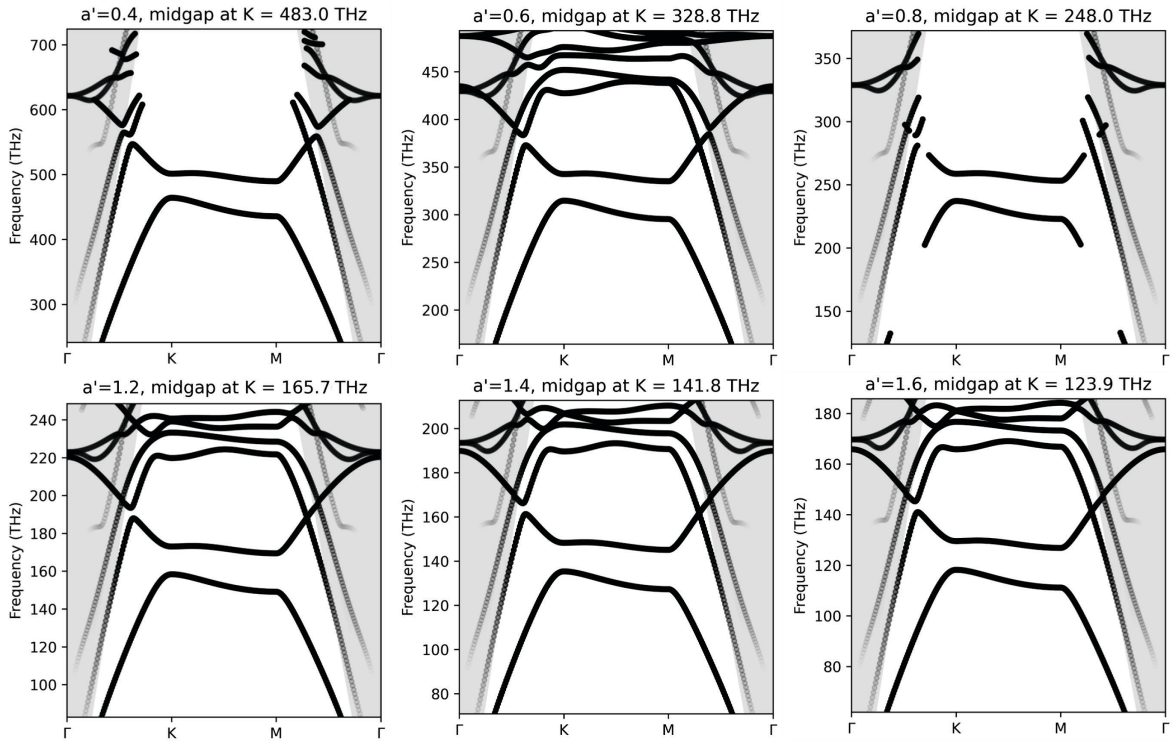

Figure S1. Band structure evolution with respect to scale parameter a'.

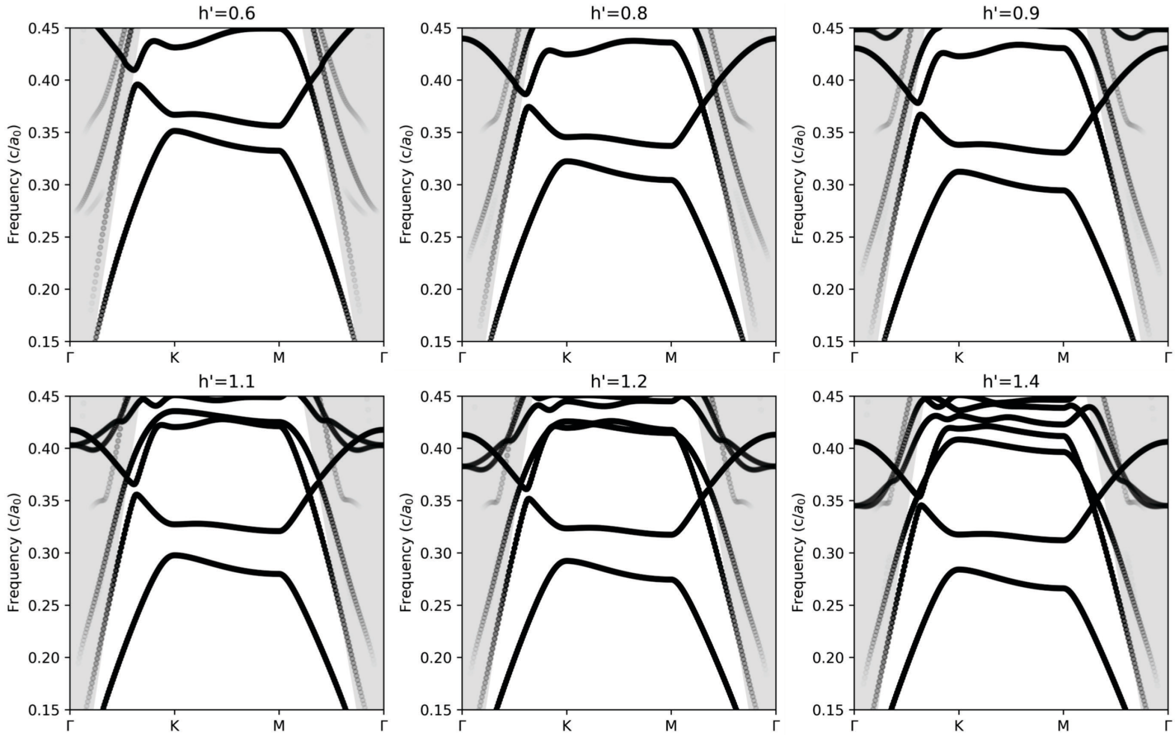

Figure S2. Band structure evolution with respect to nanopillar height parameter h'.

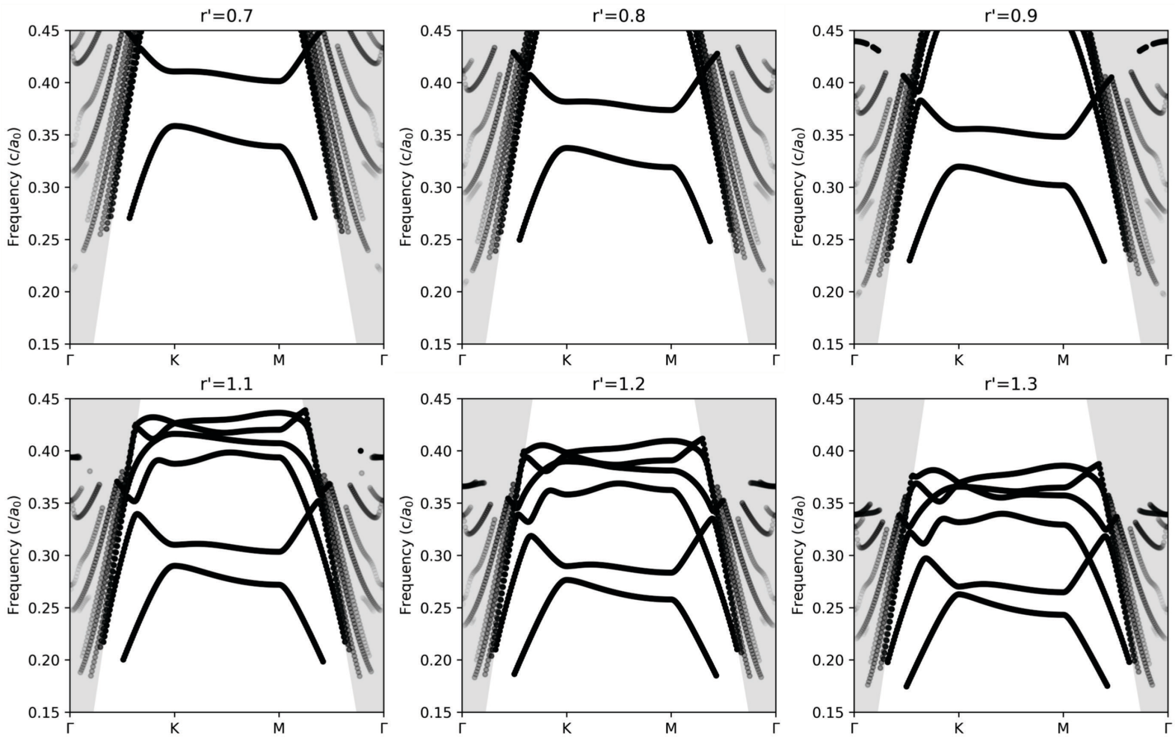

Figure S3. Band structure evolution with respect to nanopillar radius parameter r'.

2. Numerical Demonstration of the Transition Between Dielectric TM Modes and Hybrid Plasmonic Modes

To demonstrate the effect of plasmon-dielectric coupling, the field profiles of K valley states were simulated for a range of dielectric spacer thicknesses. Fig. S4(a) qualitatively illustrates the effect of plasmon-dielectric coupling, which transfers the maximum electromagnetic energy density from the silicon nanopillar to the dielectric spacer. More quantitatively, Fig. S4(b) shows the evolution of the frequencies of K valley states for several values of dielectric spacer thickness. The maximum coupling between dielectric TM modes and plasmonic-like modes takes place when energy confinement into SiO_2 is maximized, as shown on the semi-logarithmic plot of Fig. S4(c) at approximately 150 nm.

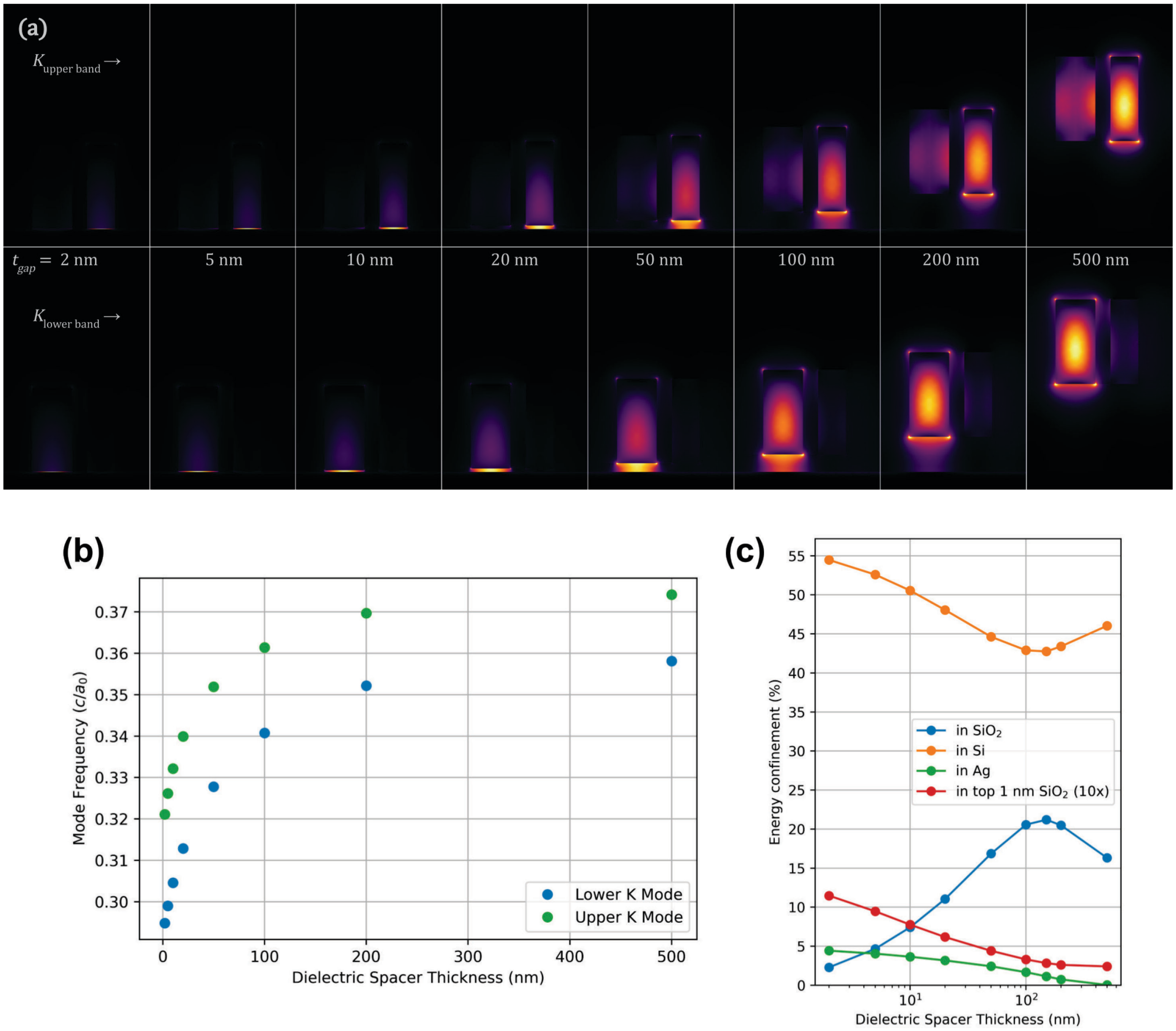

Figure S4. (a) Energy density plots of higher frequency and lower frequency K valley states demonstrate energy density enhancement with decreasing dielectric spacer thickness t_{gap}. Energy density according to Equation S1 was evaluated on the cross-sectional plane of the unit cell structure connecting the axes of nearest-neighbor expanded (left) and shrunken (right) nanopillars. (b) The frequency evolution of upper and lower K valley modes during the transition between dielectric and hybrid plasmonic modes. (c) Energy confinement in the SiO_2 spacer, the Si nanopillars, and the Ag substrate, illustrating the transition between all-dielectric modes and

plasmonic modes. Whereas the total energy confinement inside SiO_2 peaks at ~150 nm, mode energy within the remaining materials increases away from this transition point.

3. Comparison with all-dielectric and pure plasmonic topological insulators

In order to directly compare purely plasmonic, hybrid plasmonic, and all-dielectric valley-Hall structures, we designed analogous all-dielectric and purely plasmonic nanostructures and performed additional simulations to determine their propagation loss and vertical confinement. A purely plasmonic structure was designed by replacing silicon dielectric cylinders from the model nanostructure with silver, creating an array of coupled metal-insulator-metal (MIM) resonators. The parameters were changed in the following way to realize a band gap with similar frequency and bandwidth: $a_0 = 600$ nm, $h = 0.4 \cdot a_0$, and $r_{1,2} = (0.125 \pm 0.05) \cdot a_0$. Fig. S5(a) shows the physical structure and band structure of a perturbed unit cell, and Fig. S5(b) shows the corresponding supercell simulation results with edge states highlighted in red. The edge state with opposite signs of group and phase velocity was selected for analysis due to its larger bandwidth. Figure S5(c) shows the propagation loss profile of this edge state, which reaches a maximum of 0.92 dB/μm. We note that both the plasmonic losses of plasmonic and hybrid topological insulators can be slightly tuned by structural optimizations that reduce group index. However, it is observed that purely plasmonic structures experience an order of magnitude higher loss compared to results of the model hybrid valley Hall topological insulator.

All-dielectric structures are generally immune to absorptive losses due to their lack of absorbing materials. Fig. S5(d) shows the cross-sectional geometry and band structures of a silicon nanopillar valley Hall photonic crystal operating in TM polarization on an SiO_2 substrate, with

$h = 1.75 \cdot a_0$ for $\sim$10% band gap opening. Band structures for dielectric structures were also simulated via guided mode expansion using the legume package [S1]. We note that without coupling to surface plasmon oscillations, an extreme unit cell geometry containing dense features with aspect ratio above 5:1 must be leveraged to open a TM topological band gap, which poses challenges for fabrication processes. Additionally, despite the absence of absorptive losses, the presence of a dielectric substrate induces radiative losses, determined by the substrate light cone. Therefore, outside of a narrower frequency range near the lower band edge, all-dielectric structures operating in TM also experience some propagation loss, and a typical propagation loss over these wavelengths is 0.05 dB/μm.

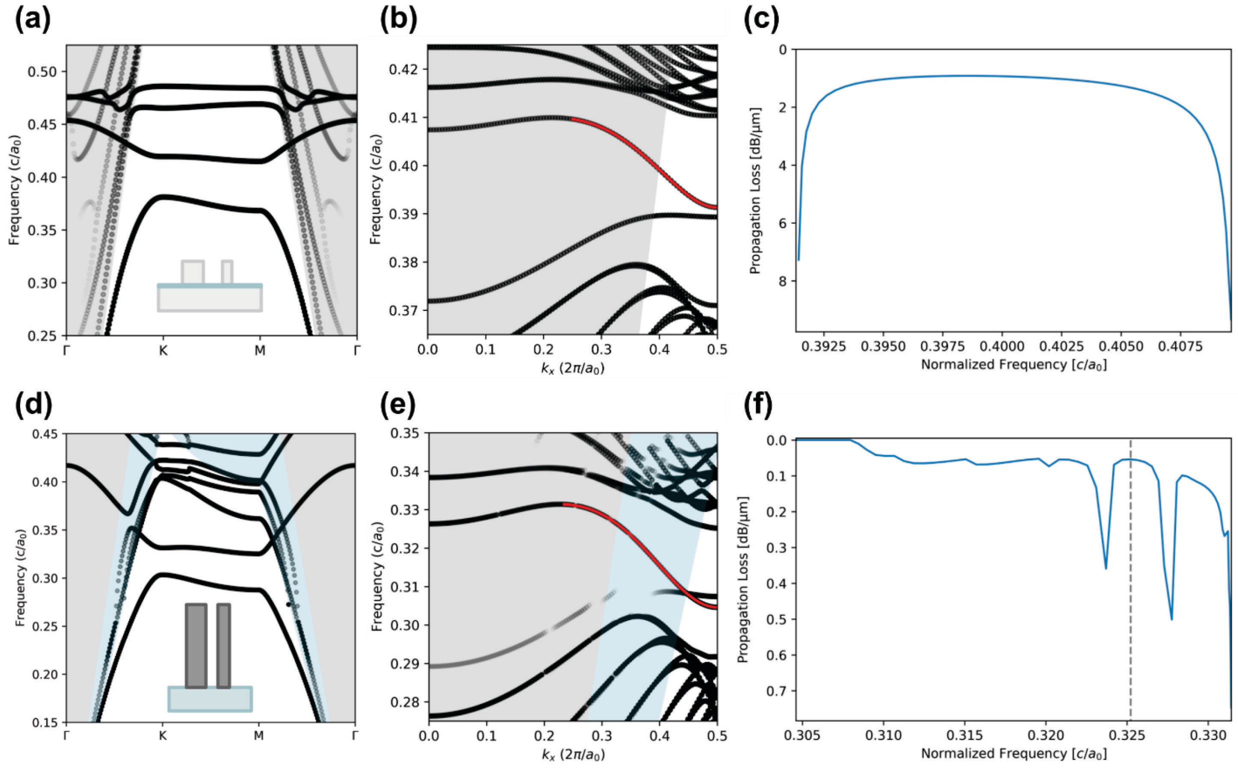

Fig. S5: Topological and propagation characteristics of purely plasmonic and all-dielectric platforms. (a, d) Geometries of both unit cells (inset) were optimized for $\sim$10% topological band gap opening, shown in the band structure. Gray regions denote the light cone, while blue regions denote the light cone in a dielectric substrate. (b, e) Within each supercell band structure, the

edge state of interest is highlighted in red. Note that the majority of the dielectric edge state lies within the substrate light cone. In (e), the TM-like modes are broken due to coupling with TE-like states, which are not shown. (c, f) Propagation loss of each edge state derived from supercell eigenmode simulation results. In (f), the grey dashed line marks the lower band edge of the upper bulk bands.

For each of these structures, the vertical confinement into a 10 nm thick volume of SiO_2 was also analyzed. For the MIM structure, 10 nm SiO_2 separates the Ag substrate and Ag pillars. For the all-dielectric structure on an SiO_2 substrate, we assume the volume of interest is a 10 nm film of SiO_2 near the substrate surface. Vertical confinement was quantified by the ratio of electromagnetic energy within the SiO_2 layer to the total modal electromagnetic energy. Energy density was evaluated as

$$W(\vec{\boldsymbol{r}}) = \frac{1}{2}\left[\frac{\partial\left(\omega\varepsilon(\omega,\vec{\boldsymbol{r}})\right)}{\partial\omega}|\boldsymbol{E}(\boldsymbol{r})|^2 + \mu|\boldsymbol{H}(\boldsymbol{r})|^2\right]. \qquad \text{(S1)}$$

The results of energy density calculations for all three classes of structures are shown in Fig. S6, and both propagation loss and energy confinement calculation results are summarized in Table S1.

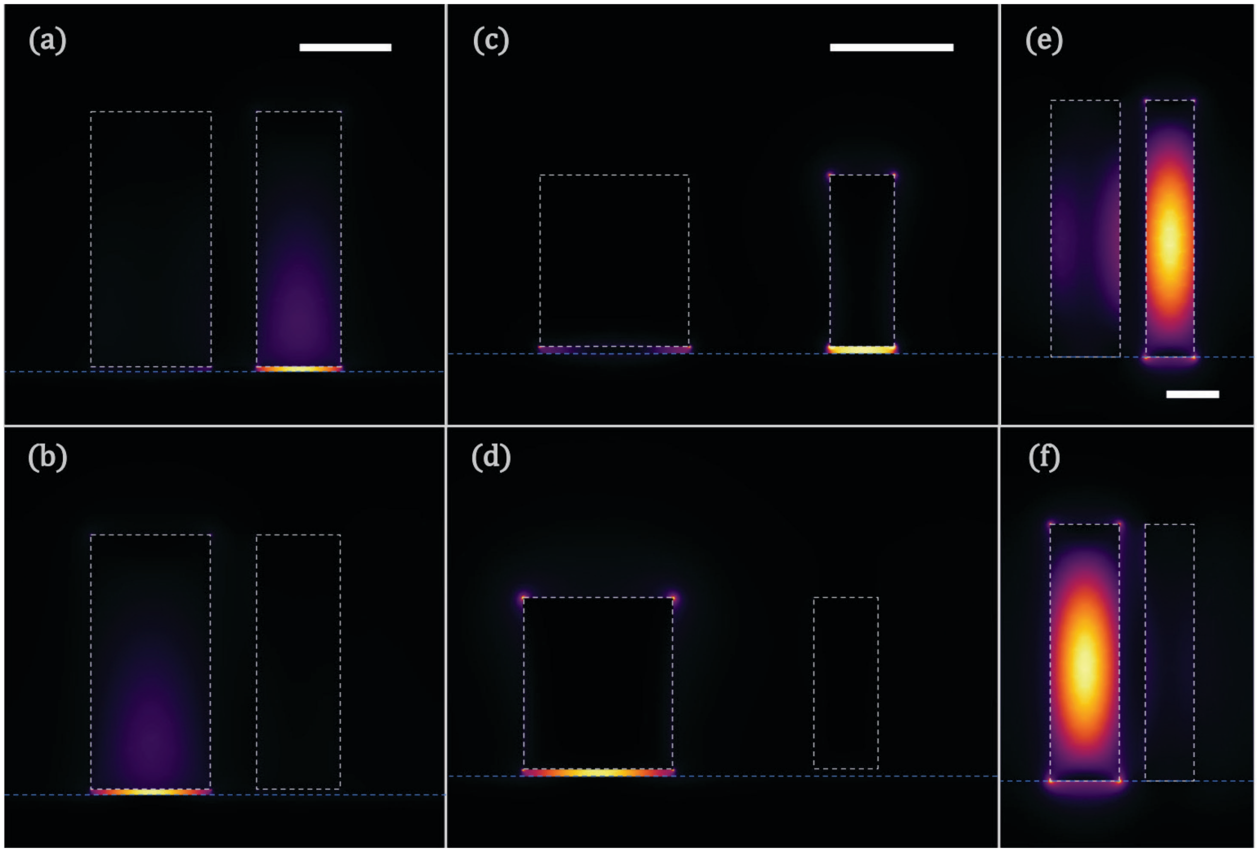

Fig. S6: Energy density cross sections of (a, b) hybrid plasmonic, (c, d) purely plasmonic and (e, f) all-dielectric valley photonic crystals evaluated for the (b, d, f) upper and (a, c, e) lower valley states. For all three material platforms, energy density according to Equation S1 was evaluated on the cross-sectional plane of the unit cell structure connecting the axes of nearest-neighbor expanded (left) and shrunken (right) nanopillars. All scale bars are 100 nm.

Table S1: A comparison of equivalent valley Hall topological insulators across material platforms.

**Property**	**All-Dielectric**	**Hybrid Plasmonic**	**Pure Plasmonic**
Energy Confinement (%)	0.54	7.3	22
Propagation Loss (dB/μm)	0 (narrowband)<br>0.05 (broadband)	0.09 (minimum)	0.92 (minimum)

In summary, hybrid plasmonic edge states achieve considerable energy confinement with some degree of propagation loss. Although purely plasmonic structures are able to confine a larger fraction of optical energy, performance gain is offset by propagation loss that is an order of magnitude larger. Despite the non-absorbing nature of dielectric structures, radiative losses into the substrate are still present at frequencies above the substrate light cone when operating in the TM polarization.

4. Analysis of Energy Confinement in Low Refractive Index Spacer

The mode energy confinement into nanoscale volumes of interest at different values of dielectric spacer thickness was calculated and analyzed according to the expression in Eqn. S1. We find that the fractional mode energy confined in SiO_2 is decreasing for gap thicknesses below approximately 150 nm, consistent with the limiting behavior of zero confinement at zero SiO_2 thickness. On the other hand, the energy confinement in the 1 nm layer immediately below nanopillars was found to be monotonically increasing down to the lowest calculated thickness of 1 nm (Fig. S7(a)). Such a metric is relevant for many technological applications, such as adsorbate sensing, carrier injection modulation, and quantum confined materials. Energy confinement for different values of spacer refractive indices were also tested, showing that fractional mode energy increases with decreasing refractive index (Fig. S7(b)).

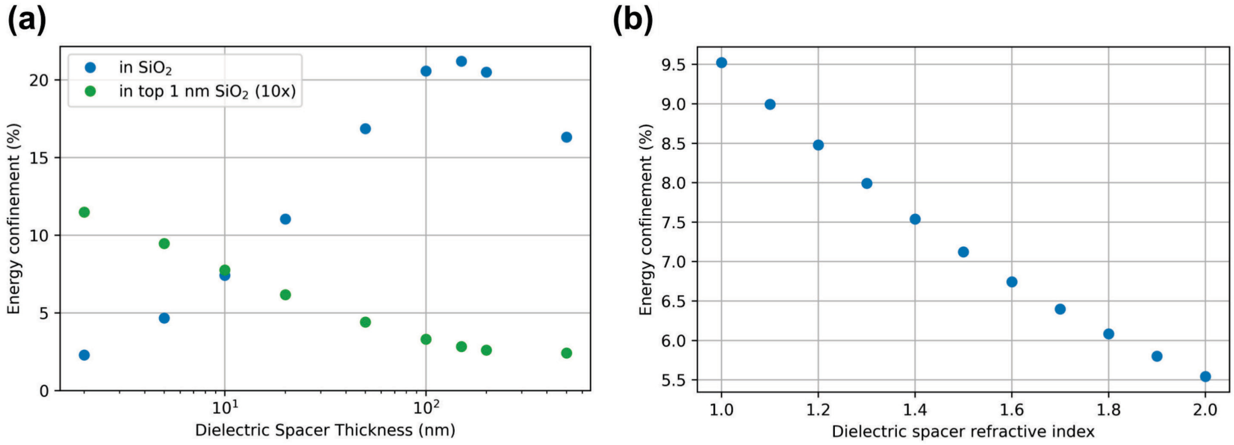

Fig. S7: (a) The 1 nm thick volume on the upper surface of SiO_2 experiences enhanced energy density down to 1 nm, while the total energy confinement within the entire SiO_2 layer is maximum at approximately 150 nm. (b) Energy confinement for increases for decreasing gap refractive index.

5. Numerical Demonstration of Chemical Sensing in Nanoscale Hybrid Plasmonic Gap Layer

Here, the application of hybrid plasmonic valley-Hall topological insulator waveguide to solution based chemical sensing is explored. We begin by modifying the model waveguide in the main text to have a refractive index of 1.42 in the dielectric gap, which should approximate that of a solvent submersed molecular imprinted polymer (MIP). The gap thickness of the waveguide was increased to 50 nm to enhance optical field energy fraction. With these parameters, the supercell dispersion was simulated with and without a refractive index change of 0.001 RIU in the MIP layer. The downward frequency shift across the edge state was approximately 0.014%, corresponding to a sensitivity of 200 nm/RIU over the telecom frequency range.

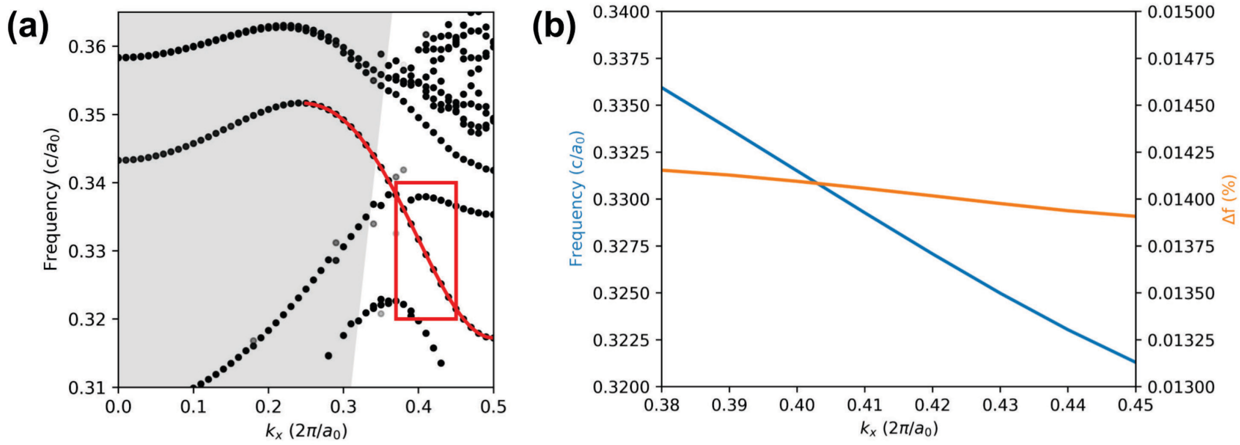

Fig. S8: (a) The supercell band structure of the model HPTI edge state modified with a 50 nm dielectric spacer with refractive index 1.42. (b) Dispersion of the HPTI edge state over the region denoted by the box in (a), and absolute fractional frequency change from 0.001 RIU modulation caused by a sensing target.

7. Discussion of real-world fabrication and characterization studies

Owing to our design process, hybrid plasmonic valley Hall topological insulators can be realized as simple structures that operate at commonly used telecommunication wavelength ranges. Thus, a wealth of methods for fabrication and characterization are available. A possible pathway to fabrication involves first depositing Ag, SiO_2, and Si layers of appropriate thickness with electron beam evaporation. Nanopillars can be patterned from the Si layer with photolithography and anisotropic dry reactive ion etching. Furthermore, silver may be exchanged for copper for full CMOS-compatibility [S2-S4]. The nanostructure proposed contains features sizes and layer thicknesses that are all easily attainable using standard nanofabrication processes, owing to the low aspect ratio of ~3 and a pattern design free of intricate geometric shapes.

To validate the 10 nm hybrid plasmonic vertical confinement of edge states, focused ion beam milling can be employed to take a cross-section of the topological waveguide, enabling access to the SiO_2 layer. Subsequently, techniques such as near-field scanning optical microscopy may be utilized to measure the field with nanometer resolution, providing direct evidence of the enhanced confinement capabilities of our proposed system [S5]. Furthermore, the topological robustness of our design can be tested. Using engineered mode couplers, standard integrated photonics waveguide characterization can be performed, and losses arising from propagation, Z-bends, and controlled disorder can be measured directly and compared. This approach not only demonstrates the structural simplicity of our design but also allows for the intuitive design of functional topological waveguide networks.

References:

[S1] Minkov, M., Williamson, I. A. D., Andreani, L. C., Gerace, D., Lou, B., Song, A. Y., Hughes, T. W., & Fan, S. Inverse Design of Photonic Crystals through Automatic Differentiation. ACS Photonics. **7**, 1729-1741 (2020). https://doi.org/10.1021/acsphotonics.0c00327

[S2] Fedyanin, D. Y., Yakubovsky, D. I., Kirtaev, R. V., Volkov, V. S., Ultralow-loss CMOS copper plasmonic waveguides. Nano Lett. 16, 362–366 (2015). https://doi.org/10.1021/acs.nanolett.5b03942

[S3] McPeak, K. M., Jayanti, S. V., Kress, S. J. P., Meyer, S., Iotti, S., Rossinelli, A., Norris, D. J. Plasmonic films can easily be better: rules and recipes. ACS Photonics. 2, 326-333 (2015). https://doi.org/10.1021/ph5004237

[S4] Chew, S. H., Gliserin, A., Choi, S., Geng, X. T., Kim, S., Hwang, W., Baek, K., Anh, N. D., Kim, Y.-J., Song, Y. M., Kim, D. E., Jeong, S.-Y., Kim, S. Large-area grain-boundary-free copper films for plasmonics. Applied Surface Science. 521, 146377 (2020). https://doi.org/10.1016/j.apsusc.2020.146377

[S5] Sorger, V. J., Ye, Z., Oulton, R. F., Wang, Y., Bartal, G., Yin, X., & Zhang, X. Experimental demonstration of low-loss optical waveguiding at deep sub-wavelength scales. Nature Communications. 2, 331 (2011). https://doi.org/10.1038/ncomms1315
